# Health Care Professionals’ and Parents’ Perspectives on the Use of AI for Pain Monitoring in the Neonatal Intensive Care Unit: Multisite Qualitative Study

**DOI:** 10.2196/51535

**Published:** 2024-02-09

**Authors:** Nicole Racine, Cheryl Chow, Lojain Hamwi, Oana Bucsea, Carol Cheng, Hang Du, Lorenzo Fabrizi, Sara Jasim, Lesley Johannsson, Laura Jones, Maria Pureza Laudiano-Dray, Judith Meek, Neelum Mistry, Vibhuti Shah, Ian Stedman, Xiaogang Wang, Rebecca Pillai Riddell

**Affiliations:** 1 School of Psychology, University of Ottawa Children's Hospital of Eastern Ontario Research Institute Ottawa, ON Canada; 2 Department of Psychology York University Toronto, ON Canada; 3 Department of Nursing Mount Sinai Hospital Toronto, ON Canada; 4 Department of Mathematics and Statistics York University Toronto, ON Canada; 5 Department of Neuroscience, Physiology and Pharmacology University College London London United Kingdom; 6 Mount Sinai Hospital Toronto, ON Canada; 7 Neonatal Care Unit University College London Hospitals London United Kingdom; 8 Department of Pediatrics Mount Sinai Hospital Toronto, ON Canada; 9 School of Public Policy and Administration York University Toronto, ON Canada

**Keywords:** pain monitoring, pain management, preterm infant, neonate, pain, infant, infants, neonates, newborn, newborns, neonatal, baby, babies, pediatric, pediatrics, preterm, premature, assessment, intensive care, NICU, neonatal intensive care unit, HCP, health care professional, health care professionals, experience, experiences, attitude, attitudes, opinion, perception, perceptions, perspective, perspectives, acceptance, adoption, willingness, artificial intelligence, AI, digital health, health technology, health technologies, interview, interviews, parent, parents

## Abstract

**Background:**

The use of artificial intelligence (AI) for pain assessment has the potential to address historical challenges in infant pain assessment. There is a dearth of information on the perceived benefits and barriers to the implementation of AI for neonatal pain monitoring in the neonatal intensive care unit (NICU) from the perspective of health care professionals (HCPs) and parents. This qualitative analysis provides novel data obtained from 2 large tertiary care hospitals in Canada and the United Kingdom.

**Objective:**

The aim of the study is to explore the perspectives of HCPs and parents regarding the use of AI for pain assessment in the NICU.

**Methods:**

In total, 20 HCPs and 20 parents of preterm infants were recruited and consented to participate from February 2020 to October 2022 in interviews asking about AI use for pain assessment in the NICU, potential benefits of the technology, and potential barriers to use.

**Results:**

The 40 participants included 20 HCPs (17 women and 3 men) with an average of 19.4 (SD 10.69) years of experience in the NICU and 20 parents (mean age 34.4, SD 5.42 years) of preterm infants who were on average 43 (SD 30.34) days old. Six themes from the perspective of HCPs were identified: regular use of technology in the NICU, concerns with regard to AI integration, the potential to improve patient care, requirements for implementation, AI as a tool for pain assessment, and ethical considerations. Seven parent themes included the potential for improved care, increased parental distress, support for parents regarding AI, the impact on parent engagement, the importance of human care, requirements for integration, and the desire for choice in its use. A consistent theme was the importance of AI as a tool to inform clinical decision-making and not replace it.

**Conclusions:**

HCPs and parents expressed generally positive sentiments about the potential use of AI for pain assessment in the NICU, with HCPs highlighting important ethical considerations. This study identifies critical methodological and ethical perspectives from key stakeholders that should be noted by any team considering the creation and implementation of AI for pain monitoring in the NICU.

## Introduction

Globally, an estimated 13.4 million babies were born preterm in 2020, accounting for about 1 in 10 of all babies born [1]. Unfortunately, a significant proportion of preterm infants require neonatal intensive care unit (NICU) due to their vulnerability to complications and health issues [2]. As part of their lifesaving care, preterm infants undergo an average of 10 to 16 painful procedures per day [3]. Unmanaged NICU pain has significant developmental consequences [4,5] and is one of the largest sources of severe emotional distress in parents [6]. Pain assessment and management is a critical aspect of care in the NICU [7]. Traditional pain assessment methods in the NICU rely on observational tools [8,9]. However, there are several challenges with these methods, including bias and subjectivity, staff time resources, and potential variability in interpretation [10-12]. Given these challenges, innovative approaches are needed to improve existing pain assessment practices. Artificial intelligence (AI), which includes machine learning (ie, using a machine to extract knowledge from data and learn autonomously), is one technology that has shown tremendous potential in the health care field, and this potential may also inform the development of clinical decision support systems [13]. Specifically, AI-based technology can analyze large volumes of behavioral, physiological, and brain imaging data to provide suggestions with regard to infant pain assessment at the point of care.

Current evidence about the use of AI in the assessment and monitoring of infant pain appears to be promising [14,15]. Preliminary algorithms to monitor vital signs [16], such as heart rate, respiratory rate, and oxygen saturation, of preterm infants have been developed, all of which provide physiological indications of pain or distress as well as systems that incorporate behavioral indicators (eg, face movements, body movements, and crying) to predict pain [17]. Although there is immense potential for these new technologies to revolutionize how neonatal pain is assessed and monitored in the NICU, a limited understanding of the perspectives of key stakeholders with regard to this emerging technology exists, that is, health care professionals (HCPs) and parents. These perspectives are essential for the successful implementation of this technology in clinical practice.

Studies exploring the attitudes and trust of clinicians toward AI in health care found that while there is recognition of AI’s potential benefits, concerns persist about reliability, transparency, data privacy, potential loss of autonomy in decision-making, and potential misinterpretation [18-21]. Factors such as age, education level, and previous experience with AI influenced attitudes and trust in AI technologies [21].

There is a growing interest in the application of AI technologies in health care, particularly in neonatal and pediatric care [14]. However, little is known about the perspectives of HCPs and parents on the use of AI for pain assessment in the NICU. Pain is a significantly different context warranting focused study because infants cannot verbalize for themselves. This study explores the perspectives of health care professionals and parents with regard to automated pain assessment using AI technology in the NICU. This study will inform the implementation of AI, specifically machine learning technology in the NICU, leading to more effective pain assessment and management strategies.

## Methods

### Ethical Considerations

Ethics approval for this qualitative study was granted from all study sites, including York University (2020-034), Mount Sinai Hospital (MSH; 19-0252-A), and University College London Hospital (UCLH; 11/LO/0350). Informed consent was obtained from all participants. All data were deidentified. Individuals were provided with a CAD $10 (approximately US $7) gift card to a local coffee shop for their participation.

### Setting and Design

Data collection occurred at 2 tertiary care NICUs: MSH (Toronto, Canada) and UCLH (London, United Kingdom). The study is part of a larger project focused on the use of AI, specifically the development of a machine learning algorithm, to assess infant pain in the NICU. Participants consisted of 20 HCPs (nurses, physicians, and allied health professionals) and 20 parents (mothers and fathers). Recruitment at MSH took place from February to March 2020, and recruitment at UCLH took place from July 2021 to October 2022. Interviews at MSH occurred in person at the hospital, whereas interviews at UCLH were web-based and conducted using a secure Zoom platform (Zoom Video Communications). This difference was due to the onset of the COVID-19 pandemic after the study had launched, which delayed the UK interviews and necessitated the use of a secure web platform. For HCPs, eligibility criteria were (1) currently providing care to infants at one of the NICUs and (2) trained as either a nurse, physician, or other health professionals (ie, outreach staff and consultant practice educator). For parents, eligibility criteria included being 18 years and older of age, having an infant who was currently receiving care in the NICU, and being fluent in English, orally (in order to respond to complex questions in the interview). Using a purposive sampling approach, all participants were initially approached by 1 clinical member of staff on the unit and asked if they were interested in participating in the study. Only families where the parent was at least 18 years of age and spoke English were approached. If interested, they received additional information, and a time was scheduled for an interview.

Following introductions and the completion of the consent form, 30-minute semistructured interviews were conducted by a member of the research team (NR, C Chow, and L Johannsson) in a private clinic room (MSH) or web-based room (UCLH). Baseline demographic information was collected at the outset of the meeting followed by a series of questions (10 for HCPs and 9 for parents) pertaining to the use of AI to inform NICU decision-making related to the assessment of infant pain. Notes were taken during the interviews to supplement transcripts. Interviewers read an initial script providing a definition of AI and providing context for the study. In-person interviews were recorded using a digital audio recorder, whereas web-based interviews were recorded using privacy-compliant web software (Zoom) and stored on a secure server. All participants were debriefed following the interview and provided with a gift card to a local coffee shop as a token of appreciation. Standards for Reporting Qualitative Research were followed for this study (Multimedia Appendix 1 [22]).

### Development of the Interview Guides

Using a grounded theory approach [23], the goal of the qualitative interviews was to generate detailed knowledge about HCPs’ and parents’ understandings and perceptions of the use of AI in the NICU to assist with infant pain assessment and management. Specifically, we sought to gain insight into HCPs’ and parents’ understanding of AI, perceived implications of this technology, potential benefits of the technology, and barriers to its use in the NICU setting. Two interview guides were developed to address the diverse perspectives of HCPs (Multimedia Appendix 2) and parents (Multimedia Appendix 3). The interview guides were developed collaboratively by members of the research team (RPR and NR), who are clinical psychologists with previous experience in conducting qualitative research with both HCPs and parents in the NICU and other pediatric medical settings [24,25]. The guides were reviewed and edited based on the feedback from team members with NICU clinical expertise (VS, C Chow, JM, and MPL-D) as well as ethical or legal or social expertise related to AI (IS). Interviews were conducted by 2 postdoctoral fellows (NR and C Chow) and 1 research staff (L Johannsson). A decision was made in advance to review and make necessary changes to the questions after the first interviews were conducted at each site based on participant comprehension and feedback. Based on the review, no major alterations were required. Participants had the opportunity to provide any additional comments or feedback at the end of the interview. Interviews were conducted until saturation was reached [26].

### Data Processing and Analysis

The interview audio recordings were anonymized and transcribed by 1 research assistant and independently double-checked by members of the research team. Transcripts were subsequently analyzed using 6 phases of thematic analysis (ie, familiarization, generating codes, identifying themes, reviewing themes, naming themes, and report writing) [27]. Data analyses took place from February to April 2023. There were 3 analysis leads (NR, C Chow, and RPR) who took primary responsibility for developing the code book, overseeing the coding process, and developing themes based on the codes generated. As a first step, the analysis leads familiarized themselves with the data by reading and making notes on the transcripts. Responses were examined for differences between the 2 sites (eg, unique considerations related to the country, time, or modality via in-person vs web-based) or any effects that may have necessitated a different analysis pathway. It was determined that there were no differences, and we proceeded with analyzing the transcripts together. Next, a list of initial codes was generated independently by the analysis leads prior to a consensus meeting. Two consensus meetings were held, where all codes were reviewed and agreed upon. Subsequently, the analysis leads (NR, RPR, and C Chow) ran a 90-minute training session with 10 coders to familiarize them with the codes that have been created. All coders (LH, SJ, OB, VS, MPL-D, C Cheng, IS, HD, NM, and L Jones) were members of an interdisciplinary research team (ie, neurobiology, behavioral neuroscience, neurophysiology, psychology, medicine, nursing, and law) with research backgrounds in pediatric health care, with most specializing in infant care. Each transcript was coded twice. The average percent agreement (ie, the number of times 2 individuals agreed upon a code divided by the total number of units of observation that were rated) across transcripts between coders for the HCP and parent transcripts was 0.77, which is adequate [28]. Next, the analysis leads reviewed the coded transcripts and collated codes for each question. The analysis leads met and generated relevant potential themes and a thematic map based on the data. Finally, examples were selected to accompany each theme, which are presented in the results below. Summary statistics of all demographic variables were conducted in SPSS (version 28; IBM Corp).

## Results

### Participant Characteristics

The participant characteristics are shown in Table 1. In total, 90% (n=18) of HCPs were university-educated and had extensive experience in the NICU (mean 19.4, SD 10.69 years; range 4-37 years). For HCPs, 55% (n=11) reported “Western” cultural heritages (eg, Canadian, British, and Australian), 5% (n=1) African, 15% (n=3) East Asian, 10% (n=2) Caribbean, 10% (n=2) South Asian, and 5% (n=1) not reported. For parents, 80% (n=16) reported “Western” cultural heritages (eg, Canadian, European, or Australian), 5% (n=1) Asian, 5% (n=1) Middle Eastern, and 10% (n=2) not reported. Most parents who participated across both sites were mothers (n=17, 85%) with a mean age of 34 (SD 5.42) years. In total, 90% (n=18) of parents had a university education or higher.

**Table 1:** Participant demographic characteristics.

Characteristics		Health care providers (n=10 each)		Parents (n=10 each)	
		Mount Sinai Hospital	University College London Hospital	Mount Sinai Hospital	University College London Hospital
**Gender, n (%)**					
	Women	9 (90)	8 (80)	8 (80)	9 (90)
	Men	1 (10)	2 (20)	2 (20)	1 (10)
Age (years), mean (SD)		—^a^	—	34.56 (6.04)	34.2 (5.14)
Postnatal age of infant (days), mean (SD)		—	—	28.11 (24.09)	57.5 (29.52)
**Highest level of education, n (%)**					
	Graduate school or professional training	6 (60)	7 (70)	3 (30)	9 (90)
	University graduate	2 (20)	3 (30)	5 (50)	1 (10)
	Partial university	0 (0)	0 (0)	0 (0)	0 (0)
	Trade school or community college	1 (10)	0 (0)	1 (10)	0 (0)
	High school graduate	0 (0)	0 (0)	0 (0)	0 (0)
	Less than high school	0 (0)	0 (0)	1 (10)	0 (0)
	Not reported	1 (10)	0 (0)	0 (0)	0 (0)
**Heritage culture, n (%)**					
	African	1 (10)	0 (0)	0 (0)	0 (0)
	Asian	2 (20)	1 (10)	1 (10)	0 (0)
	Australia or New Zealand	0 (0)	0 (0)	0 (0)	2 (20)
	Caribbean	2 (20)	0 (0)	0 (0)	0 (0)
	Canadian	1 (10)	0 (0)	5 (50)	0 (0)
	European	3 (30)	7 (70)	1 (10)	8 (80)
	Middle Eastern	0 (0)	0 (0)	1 (10)	0 (0)
	South Asian	0 (0)	2 (20)	0 (0)	0 (0)
	Not reported	1 (10)	0 (0)	2 (20)	0 (0)
**Type of health care professional, n (%)**					
	Physician	5 (50)	3 (30)	—	—
	Registered nurse	5 (50)	4 (40)	—	—
	Other health professional	0 (0)	3 (30)	—	—
Experience (years), mean (SD)		22 (8.55)	16 (12.18)	—	—

^a^Not available.

### HCP Themes

**Figure 1:**
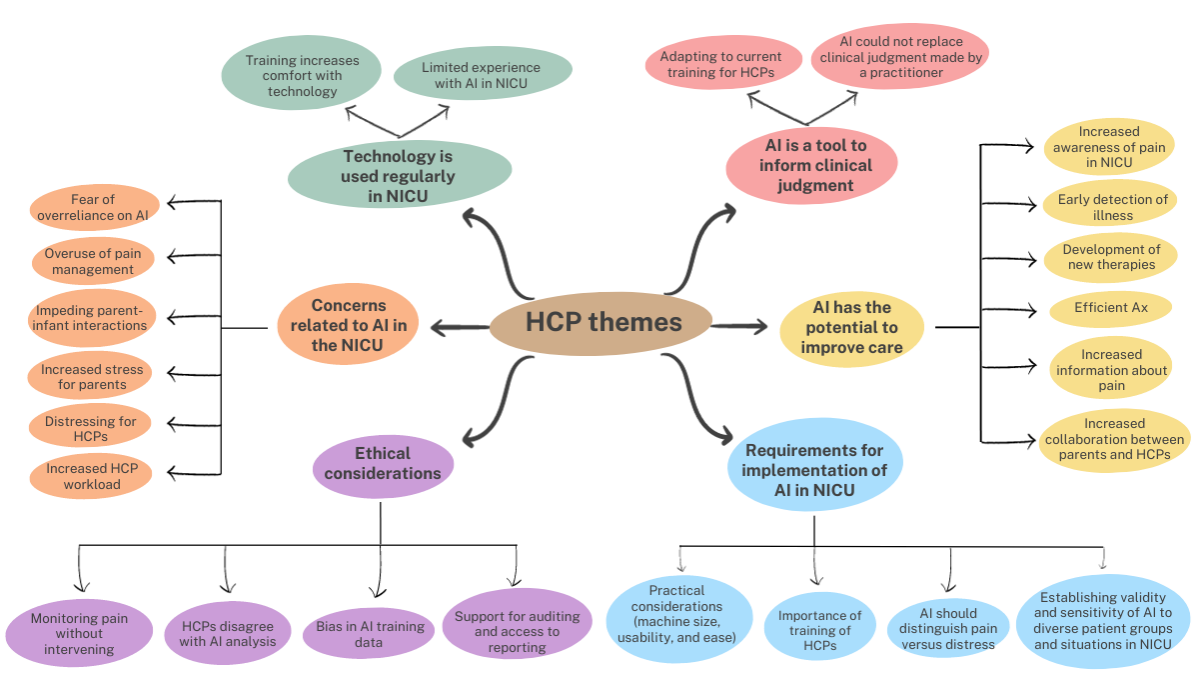
Themes and subthemes generated from qualitative interviews with HCPs on their perspectives about using AI to assess pain in the NICU. AI: artificial intelligence; Ax: assessment; HCP: health care professional; NICU: neonatal intensive care unit.

Six themes emerged from the thematic analysis on the HCP interviews. Each theme, a description, and representative quotes are presented in Table 2. HCP themes and subthemes are presented in Figure 1. First, in the context of their comfort with incorporating new AI technology, HCPs reported limited experience with AI technology in the NICU (1 HCP was part of a research study at another institution), and they were comfortable using other forms of technology. Second, HCPs identified some concerns with regard to the integration of AI for pain assessment in the NICU. Some of these concerns included increased distress from knowing clinicians were inflicting pain and extra workload for HCPs, increased stress for parents, and decreased opportunities for parent-child bonding, as well as fears related to overreliance on AI technology and the overuse of medication to manage pain. Despite these concerns, the third theme emerged surrounding several benefits that AI could bring to the NICU context. Notably, HCPs identified increased awareness of infant pain, early detection and diagnosis of clinical changes, increased efficiency, and standardization of pain assessment, as well as the potential to inform the development of better pain management strategies. From a practical standpoint, the fourth theme identified requirements to facilitate the implementation of AI in the NICU, including the size of machinery, staff training, as well as clearly communicating the validity, sensitivity, and specificity of the algorithm being used. The fifth theme that was unanimously shared was the idea that using AI for pain assessment in the NICU would be a tool for HCPs to use but could not replace the clinical judgment and decision-making of an HCP. Concerns related to how the next generation of HCPs would be trained to ensure that they have both the clinical and technological skills to operate in the NICU were described, given the potential overreliance on technology. Finally, HCPs identified the potential for ethical concerns related to an AI algorithm for constant pain monitoring in the NICU, specifically, issues related to the disagreement between HCP and the AI algorithm, implications of pain monitoring in the absence of pain management, as well as the need to audit the algorithm. Overall, there was general acceptability for the benefits, use, and integration of AI technology for pain assessment in the NICU, with keen identification of the potential work-related, structural, technological, and ethical issues that would need to be addressed to facilitate implementation.

**Table 2:** Key themes identified by HCPs^a^ with regard to the use and integration of AI^b^ for pain assessment in the NICU^c^.

Theme	Description	Representative quote
Technology is used regularly in the NICU	HCPs shared that despite having limited experience with AI specifically, they use technology to inform their clinical decision-making and they feel comfortable using the technology that is currently available.	“It informs everything. I think that’s one of the things that working in intensive care is that we use technology and monitoring to inform a lot of our decisions.”
Concerns of AI integration for pain assessment in the NICU	HCPs identified concerns related to the integration of AI in the NICU. It specifically increased the workload for HCPs and increased distress, knowing they were potentially inflicting pain on an infant. They also reported that constant pain monitoring could increase stress for parents and that added machinery could inhibit parent-child bonding. Concerns were also identified with regard to the overreliance on what the algorithm reported and the overuse of pain pharmaceuticals to manage pain.	Increased HCP distress: “I’m not sure cause you imagine like how upsetting it would be like you know I’m doing a diaper change and this thing is telling me the baby is in pain.” Increased workload: “I think there would be some negative feedback towards having extra work to be done.” Fear of overreliance on the AI: “The disadvantages would be that we become over reliant on it. And just because the machine says the baby’s not in pain, then it could be dismissed as the baby isn’t in pain, when actually if you look at the baby, you can tell they’re in pain.” Increased parent stress: “It can cause stress ... Unnecessary stress.” Impeding parent-child bonding: “I can see it taking away from looking at babies...you see parents, particularly looking at their monitor alarms, for whatever reason, they look more at the monitor than actually what their baby’s doing.”
AI has the potential to improve pain assessment and management	HCPs indicated there are several ways in which integrating constant pain monitoring in the NICU could improve clinical care, including the development of new therapies, early diagnosis of difficulties, detection of changes in clinical presentation, increased awareness of infant pain, increased efficiency of pain assessment, increased standardization of pain assessment, and increased collaboration between HCPs and parents.	“I think it’s good that um there is a form of technology that can give us more information about pain in this population because I think there’s a lot of unknown and I think well I know for myself like I said I can’t honestly say that I’m always thinking about if this baby is in pain or what kind of pain this baby is in when doing a procedure.” “I think it would give them more time to obviously focus on other aspects of their work instead of having to score every half an hour or so to proceed and enter the data as it is at the moment.”
Requirements for implementation of AI in NICU	HCPs described structural (ie, machine size and invasiveness of machinery) requirements for implementing AI in the NICU. Specifically, machinery would need to be small and noninvasive. HCPs indicated that training staff to understand and interpret the output provided by the technology is important. They also indicated that the algorithm would need to be properly validated and sensitive for detecting pain in diverse patient groups and situations.	Structural requirements: “It depends how invasive the technology is. When you have a 450 gram baby in front of you. Even putting on things like more monitors actually occludes your that visual assessment of the child. So I think there can be barriers.” Importance of training: “I think obviously, it’s all about training ... everybody understands how it works and the benefits.”
AI is a tool to inform clinical pain assessment and management	HCPs indicated that AI in the NICU should be viewed as a tool to inform clinical decision-making but not as a replacement. They also indicated that the integration of this technology would have implications for the training of new HCPs to ensure they have the ability to understand how this tool could inform their own clinical assessment.	“I like using technology but as long as it doesn’t replace my ability to provide comfort and care” “If I’m gonna make it’s just detection of pain, I think it’d be fairly comfortable with that. Because then I can react to that. Whereas if it’s making medical decision on the treatment, a baby’s receiving, I think that will be a completely different scenario.”
Ethical concerns with constant pain monitoring may occur	HCP indicated the need to be aware of ethical concerns like the potential bias in AI algorithms, disagreements between HCPs and the AI’s output, and the implications of constant pain monitoring without intervening. HCPs also indicated that algorithms would need to be audited and monitored over time.	“And then you have to decide, what you want to do about it. And then you have to decide, in a medical-legal issue whether to believe A.I. or the clinician and that will be interesting.”

^a^HCP: health care professional.

^b^AI: artificial intelligence.

^c^NICU: neonatal intensive care unit.

### Parent Themes

**Figure 2:**
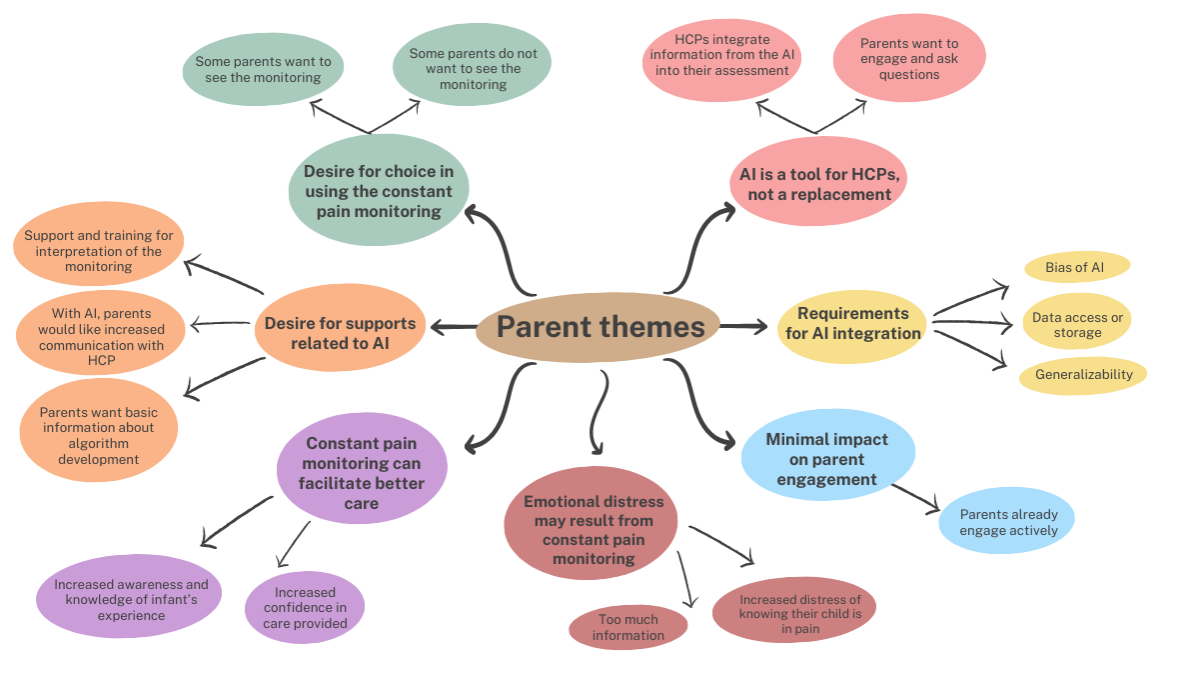
Themes and subthemes generated from qualitative interviews with parents on their perspectives of using AI to assess pain in the neonatal intensive care unit. AI: artificial intelligence; HCP: health care professional.

Seven overarching themes were identified with parents (Table 3). Parent themes and subthemes are presented in Figure 2. First, parents indicated it would be desirable to know if their infants were in pain because there are limited ways of assessing neonatal pain and it would provide useful information to HCPs to improve their infant’s care. However, the second theme arose about the emotional toll that may be experienced by parents. Some parents noted heightened distress from knowing their infant was experiencing pain. The third theme revolved around a preference to have parents decide for themselves whether they wanted continuous pain monitoring using AI. The fourth theme was that parents indicated wanting support to interpret and understand the constant pain monitoring. That is, they would want HCPs to explain their decision-making process as well as how the pain assessment provided by the AI was being used. The fifth theme was that parents perceived their current level of engagement in their infant’s care to be quite high and they did not think constant pain monitoring would change this engagement. The sixth theme was that most parents would not trust an AI to make an independent decision about their infant’s pain but rather believe it should be incorporated as a tool by HCPs to make a clinical decision. Parents voiced that there would be potential for error in the AI’s assessment and that verification by an HCP would be important. Finally, parents identified requirements related to AI integration in the NICU. Specifically, they are concerned about privacy since large amounts of data would be collected and therefore would need to be kept secure. They also identified that the algorithm should be developed in a nonbiased way and that generalizability of the algorithm across infant presentations and contexts would be needed.

**Table 3:** Key themes identified by parents with regard to the use and integration of AI^a^ for pain assessment in the NICU^b^.

Theme	Description	Representative quote
Constant pain monitoring can facilitate better care	Parents indicated there are advantages to constant pain monitoring (eg, increase in awareness of infant’s experience and confidence in care provided).	“But then it could also help the parent, could help us understand the baby a bit more and maybe bond maybe a bit more or communicate in a way with the baby more.”
Emotional distress may result from constant pain monitoring	Parents shared disadvantages to constant pain monitoring, such as too much information or distress associated with knowing their child is in pain.	“My gut is saying, as a parent, well, of course. But I’m wondering whether you can have almost too much information, where if certain things, I definitely would be in this position, where if certain things had to be done to my child, life and death or even just less serious, but they needed to be done for, you know, health reasons, how productive is it for a parent to know exactly how much pain their child is in.”
Desire for choice in using the constant pain monitoring	Parents indicated that they would like to be given a choice to view the constant pain monitoring.	“You should have a choice in the same way as like, you can choose to look at lots of the information about your baby or not.” “I mean I would want to know if my baby is in pain or not. But maybe some parents are ok with or don’t want to know about their baby’s pain but to me I would definitely want to see.”
Desire for support related to AI	Parents indicated that they would want communication from staff and support to understand and interpret the constant pain monitoring. They would also like basic information about how the algorithm was developed and makes its predictions.	“Because even now I don’t want to do anything unless the nurse is there ... but you see that number go up as you’re as you’re caring for the baby you might be or I might be a little apprehensive um but with the reassurance of the nurse or if you can see that once the baby is settled down the baby is more comfortable again then you know that it’s ok.” “I would want to know, and I would want it to be very clear why those decisions were made. I would want, if we were using kind of artificial intelligence, what kind of almost a report on why those decisions were made and why it was recommended that XYZ happened as a result.”
Minimal impact on parent engagement	Parents indicated that constant pain monitoring would minimally impact their level of active engagement in the newborn’s care as most reported that they already engaged at a high level.	“You know I’m not sure that it would change how engaged I would be because I think you know you can use other metrics as like surrogate of pain as well and being at the bedside you can still be engaged in her care but I guess it could be interested to ask you know like when we should up like you know how were her pain scores overnight or something like that. And you know get that data and get that information from the bedside nurse. But I don’t think it would dramatically change the engagement.”
AI for pain monitoring is a tool for HCPs^c^, not a replacement	Parents indicated that constant pain monitoring should be used as a tool to inform clinical judgment.	“Yeah no I would like the doctor so I could also ask questions and you know it’s yeah a tool to assess or to inform them” “And I think it makes sense that the physician in the bedside needs to integrate that with what their clinical assessment is” “It would be a good thing if doctors were checking in to validate that the AI was right and if they disagree they should definitely question it [...] maybe the model is wrong or like maybe the model just needs to be tweaked and it needs doctors and scientists to question it right? It’s probably a good thing.”
Requirements for AI integration in the NICU	Parents indicated that it would be important to consider how data might be collected and used by the AI, how to reduce bias in the development of the algorithm, and how to ensure that the algorithm was generalizable across infants and contexts.	“... questions about the data it was collecting and where that was going and who’s using that data. So obviously, the monitoring there’s a lot of information there.” “I would be concerned if a model was created that the way in which it was created was maybe not ethical but I’m I know there’s all kinds of laws and things like that but I was just thinking about how that might work.” “And then the sample size and the how many different like every baby is different and every baby’s pain tolerance is different how do you know that you’ve got all your bases covered for all the different scenarios.”

^a^AI: artificial intelligence.

^b^NICU: neonatal intensive care unit.

^c^HCP: health care professional.

## Discussion

### Principal Findings

This international study includes the perspectives of both HCPs (ie, physicians and nurses) and parents regarding the use of AI technology in the NICU setting. These perspectives offer critical insights to help inform the development of potential AI technology on infant pain management and integration of this technology as part of clinical decision support systems. We found that both HCPs and parents were supportive of the use of AI technology in predicting infant pain. Both HCPs and parents recognized that AI has the potential to improve care in the NICU setting. Other studies have also identified similar benefits including earlier detection of illness, increased collaboration and communication, and development of new treatments that further support the use of AI in clinical settings [29,30].

In line with previous research [31], this study also found that HCPs and parents had similar concerns on the use of AI technologies in the NICU setting, including effectiveness and accuracy, fear of overreliance, and shared decision-making over the use of AI technology. Furthermore, we identified additional themes from the perspectives of parents regarding the importance of receiving support for interpreting and understanding constant pain monitoring. Interestingly, most parents indicated that they would prefer the choice to have access to constant pain monitoring in real time, as it could impact parents differently. Moreover, both HCPs and parents identified the importance of using AI as an adjunctive tool to inform clinical decisions. That is, both parents and HCPs seemed in favor of using AI to augment human intelligence and support more informed clinical decision-making [32] rather than automating any aspect of clinical care. Similar to youth and adult patients, parents of infants in the NICU were concerned about the risk of clinician replacements and emphasized the importance of the human element (ie, HCP’s presence at the bedside) in clinical care [30,33,34]. Clinicians also warned about the potential for diminished skills and overreliance on technology for the next generation of clinicians with regard to pain assessment at the bedside. It is worth noting that clinical decision-making and responsibility continue to rest with clinicians, and there is currently no legislation that would allow automated health care decisions by an AI [35]. These new emerging themes could potentially help inform the future development of AI tools in the NICU setting as well as the training of future HCPs working in the NICU. Findings from this study could be used to justify increased training, engagement, and consultation with health care professionals as AI is implemented in the NICU.

Interestingly, we found very similar responses and results across countries as well as interview modalities. This is not surprising as both the United Kingdom and Canada follow similar protocols within the NICUs as both have public health care systems. Additionally, structured interviews, such as those conducted in this study, work equally well in face-to-face or web-based studies [36]. Furthermore, the interviewers were the same across both contexts. We also found that both HCPs and parents had limited experience with the use of AI in the NICU, meaning that all the responses garnered in this study were hypothetical in nature. Had participants had exposure, they may have provided different responses with regard to the feasibility and use of this technology. Future research prior to and during the implementation process will be important to capture these perspectives.

### Limitations

There are some limitations to this study that should be considered when interpreting our results. First, interviews were conducted with HCPs at 2 large, tertiary-care, academic hospitals in Canada and the United Kingdom that are at the forefront of technological advancement in the NICU. As such, the perspectives of HCPs in this study may not be generalizable to smaller, less well-resourced care settings. Second, parents included in this study were highly educated, which may limit generalizability to parents with lower educational attainment, which is also a known risk factor for preterm birth [37]. Moreover, parents were recruited into the sample if they spoke English, which may have resulted in a less culturally diverse sample. Third, many of the themes that were identified by HCPs and caregivers were broad in that they were not referring to the use of AI specifically but rather the use of clinical decision support systems (ie, a clinician using technology like AI to help inform their decisions related to care). As both technology and terminology evolve in the medical context, it will be important to disentangle opinions related to the technology itself as opposed to its use as a clinical decision-making tool. Finally, questions asked of HCPs and parents differed with more emphasis placed on general technology with HCPs and on neonatal pain for parents. This may have had an impact on the responses that were generated. As AI-related technology is integrated into medical settings, future qualitative research may focus specifically on pain-related questions.

### Conclusions

Based on detailed interviews with 40 HCPs and parents across 2 large NICUs in publicly funded hospitals in Canada and the United Kingdom, our overall findings indicate that both HCPs and parents view the integration of an AI algorithm for constant pain monitoring to have potential benefits and to be an acceptable practice. Notably, HCPs identified several ways in which constant pain monitoring could improve the clinical care provided in the NICU. Both HCPs and parents were balanced in their perspectives and identified potential disadvantages as well as requirements for the successful implementation of an AI tool for pain assessment. Taken together, there is immense promise as well as major structural, ethical, and methodological considerations for the development and implementation of AI technology in the NICU setting.

## Appendix

Multimedia Appendix 1 Standards of Reporting Qualitative Research [22].

Multimedia Appendix 2 Interview guide for health care professionals.

Multimedia Appendix 3 Interview guide for caregivers.

## Abbreviations

AI: artificial intelligence
HCP: health care professional
MSH: Mount Sinai Hospital
NICU: neonatal intensive care unit
UCLH: University College London Hospital
