# Supplementary material for: Health Care Professionals’ and Parents’ Perspectives on the Use of AI for Pain Monitoring in the Neonatal Intensive Care Unit: Multisite Qualitative Study
Source: JMIR AI. 2024 Feb 9;3:e51535. doi: 10.2196/51535 (PMC11041412; doi:10.2196/51535)
Supplement: Multimedia Appendix 2 [file ai_v3i1e51535_app2.docx]

**Introductory Script:** Decades of research have shown that pain assessment of preterm infants in the NICU is a complex and challenging task. Interviews that we have conducted with health care providers have told us that nurses and physicians use many different indicators to determine whether a preterm infant is in pain and whether an intervention should be used. Some of these indicators include an inability to settle, grimacing, a tense body, dysregulated sleep patterns, oxygen desaturation, or irregular heart rate. We can also use information about pain processes in the brain using electrical potentials that can be monitored through electrodes on your infant’s scalp (EEG). One of the biggest challenges in using these different indicators is that it is difficult to know which one to prioritize or whether patterns of indicators are identified that can accurately notify whether an infant is experiencing pain or not. Integrating and keeping track of thousands of bits of information simultaneously and trying to identify patterns and make decisions is beyond human capability to do.

Working with an international team of leaders in infant pain and distress and leading computer and mathematical scientists, we are setting out to discover what specific patterns of heart rate, brain electric potentials, oxygen saturation levels and facial grimacing could tell us whether an infant is in distress from pain from a heel lance, in distress for reasons other than a medical procedure (e.g., hunger) or no pain at all. We will be using mathematical modelling and machine learning techniques. In essence, we would be building a program that would identify patterns of pain indicators that could eventually help us determine whether or not the infant is in pain. The hope would be that this technology could be used to help inform more accurate and helpful clinical decision making in the NICU, leaving open the potential for bedside monitoring – just like heart rate or oxygen saturation.

An important part of developing this technology and eventually incorporating it into the clinical setting involves input and perspective from health care providers like you. We appreciate you taking the time to answer some of our questions.

Start with an introductory question about the healthcare provider’s role.

1. Please discuss your comfort level with the use of technology in healthcare decision making?
2. Do you have any exposure to the use of machine-based algorithms within the health care setting?
3. Do you foresee any barriers or challenges to the implementation of this type of ‘pain monitoring technology’ in your clinical setting?
4. Do you foresee advantages or disadvantages to the implementation of this type of ‘pain monitoring technology’?
5. Do you think this technology should be used and, if yes, how?
6. What do you see are the practical changes in the care provided by physicians and nurses?
7. How much trust should be placed in a prediction made by such an algorithm? What steps should happen if a physician or a nurse does not agree with a prediction,
8. What is your comfort level with the implementation of this type of technology in your clinical setting? If not, what would make you more comfortable?
9. Do you have any ethical concerns with regards to the implementation of this type of technology in your clinical setting?
10. Machine learning algorithms need to be tested to ensure they are still accurate. Do you think it would be important to test the algorithm’s predictions on an ongoing basis and make the results of that audit publicly available? Or could the audit be a part of internal quality assurance purposes?
11. Other Thoughts?
