# Supplementary material for: Health Care Professionals’ and Parents’ Perspectives on the Use of AI for Pain Monitoring in the Neonatal Intensive Care Unit: Multisite Qualitative Study
Source: JMIR AI. 2024 Feb 9;3:e51535. doi: 10.2196/51535 (PMC11041412; doi:10.2196/51535)
Supplement: Multimedia Appendix 3 [file ai_v3i1e51535_app3.docx]

**Introductory Script:** Preterm infants go through many painful procedures each day. Identifying and managing this pain is important for infant’s short- and long-term outcomes. Because preterm infants cannot tell us directly that they are experiencing pain, we need to find new ways to assess their pain. We believe using artificial intelligence can make pain assessment so much better for babies born preterm.

Based on decades of research, our team has learned that there are many different signals or clues that tell us when preterm infants might be in pain. For example, we can look at their heart rate, their blood oxygen saturation, their facial expressions, and pain signals that are happening in their brains. However, we don’t know which of these signals to pay most attention to or whether a combination of these signals may be the most effective way to understand whether or not a child is in pain. In a busy NICU, it is impossible for health care providers to measure and analyze all of this information and to then make a clinical decision. However, with a computer, we believe that we could provide a tool to health professionals to help them better understand an infant’s pain.

We are conducting research to understand if there are patterns of heart rate, oxygen saturation levels, facial expressions and brain activity that can help us to know if a baby is in pain. We are using artificial intelligence, specifically machine learning to look for patterns. This is a fancy way of saying that the computer helps us to read and interpret all these different pain signals to help guide healthcare professionals on what to do next. This would mean that all these pain signals would be monitored and the healthcare provider would get real-time feedback about your infant’s pain level. This would allow them to know when to intervene for pain versus other causes that could be distressing the infant. As we develop this tool, we are very interested in having the opinions and thoughts of parents and caregivers who have preterm infants being treated in the NICU. We appreciate you taking the time to answer some of our questions.

Start with an introductory question to make the participant feel comfortable (e.g. what is your child’s name, how long have they been in the NICU)

1. Do you think parents would find it useful to have access to real-time information about their newborn’s pain levels? Do you think this information would be useful for health care providers?
2. What might be some advantages and disadvantages of having access to real-time information about your infant’s pain?
3. Do you think knowing this information would affect parental stress levels? Should parents be given the choice of seeing or not seeing the pain assessment information generated by a computer?
4. If there was constant real-time monitoring of their newborn’s pain, would this affect your confidence in the care the newborn was receiving?
5. If you had access to real-time monitoring of the newborn’s pain, would this impact your level of active engagement in the newborn’s care?
6. How much information would you want to know about how a computer made a decision about the newborn’s pain?
7. Do you trust computers to make judgements about the newborn’s pain? What about in the absence of the judgement of a healthcare provider?
8. If you knew an algorithm was involved in a health professional’s decision-making process, would this affect your level of trust in the health professional?
9. If a physician or nurse decided to make a decision that disagreed with the computer, would you want to know? How do you think these disagreements should be addressed?
